# Mapping of crown rust resistance gene *Pc53* in oat (*Avena sativa*)

**DOI:** 10.1371/journal.pone.0209105

**Published:** 2018-12-26

**Authors:** Belayneh Admassu-Yimer, J. Michael Bonman, Kathy Esvelt Klos

**Affiliations:** 1 Oak Ridge Institute for Science and Education (ORISE) Research Participant, Small Grains and Potato Germplasm Research Unit, Agricultural Research Service, United States Department of Agriculture, Aberdeen, Idaho, United States of America; 2 Small Grains and Potato Germplasm Research Unit, Agricultural Research Service, United States Department of Agriculture, Aberdeen, Idaho, United States of America; Julius Kuhn-Institut, GERMANY

## Abstract

Crown rust disease caused by the fungus *Puccinia coronata* f. sp. *avenae* (*Pca*) is a major production constraint of oat in North America, Europe, and Australia. There are over 100 genes effective against one or more *Pca* races, but only a handful of seedling resistance (*Pc*) genes have been mapped to a known chromosomal location. The goal of the present study was to use linkage mapping to identify the genomic location of the *Pc53* gene, and to produce a list of linked SNPs with potential as molecular markers for marker assisted breeding. The *Pc53* gene was placed on the linkage group Mrg08 at 82.4 cM using F_5_-derived recombinant inbred lines (RILs) from a cross between the *Pc53* carrier 6-112-1-15 (PI 311624) and the susceptible cultivar Otana. The map location was validated using RILs from a cross between 6-112-1-15 and the *Pc50* differential line. Single nucleotide polymorphism marker GMI_ES02_c14533_567 was the closest to *Pc53*. A major seedling resistance gene ‘*PcKM*’ and QTL *QcC*.*Core*.*08*.*1*, *QCr*.*Core*.*08*.*2*, *QCr*.*Core*.*08*.*3* and *QCr*.*cdl9-12D* were previously reported on Mrg08. *QPc*.*Core*.*08*.*1* and *PcKM* were mapped to within 1 cM of *Pc53*; but previous virulence studies have indicated separate identities. The chromosomal location of *Pc53* and SNPs linked with it will facilitate the utilization of *Pc53* in oat breeding programs.

## Introduction

Crown rust caused by the fungus *Puccinia coronata* f. sp. *avenae* (*Pca*) is a major production constraint of oats (*Avena sativa* L.) in North America [1]. The disease is also present in most oat producing countries of the world [2, 3]. The virulence of the pathogen evolves continuously as it undergoes sexual recombination on the alternate host buckthorn (*Rhamnus spp*.) in North America [1]. There are effective fungicides available to manage crown rust in oats, but their use may not be economically justifiable [4]. Deployment of cultivars carrying effective genes against the pathogen is generally accepted as the best strategy for managing rust diseases in small-grain cereals including oat crown rust [4–6]. Over 100 race-specific crown rust resistance genes have been identified in oats. Most of these genes originated from the wild oat species *A*. *sterilis* [7]. ‘Fidler’ was the first oat cultivar to be developed in the 1980s with the *A*. *sterilis*-derived resistance gene, *Pc39*. Since then, genes *Pc38*, *Pc39*, *Pc48*, *Pc68* and *Pc94* have been deployed in cultivars such as: Dummont, AC Medallion, AC Assiniboia, AC Pinnacle, and Leggett with widespread use [7]. Unfortunately, the effectiveness of singly deployed seedling resistance genes is short-lived [8, 9] due to the continuous evolution of virulence in the pathogen in North America [1].

*Pc53* is one of the many *Pc* genes identified in oats. It was originally identified from the wild oat species, *A*. *sterilis*, in the Mediterranean region of Israel (GRIN, http://www.ars-grin.gov/npgs/). Despite the occurrence of virulence to *Pc53*, the frequency of virulence against this gene in North America had been consistently low compared to most *Pc* genes [1, 10, 11], suggesting that it might be a useful gene to utilize in oat breeding for crown rust resistance.

Only a handful of the nearly 100 *Pc* genes have been mapped to a known chromosomal location of oat [6,12,13]. These include *Pc38* [14], *PcKM*/*Pc45* [13], *Pc58* [12,15], *Pc68* [16], *Pc71* [17], *Pc91* [6,13] and *Pc94* [18]. Absence of information about the chromosomal location of most of the *Pc* genes has created multifaceted challenges in oat research. First, it has limited the utilization of genomic tools in oat breeding. Second, in the continuous search for new sources of crown rust resistance, the lack of information on genomic location restricts investigations as to novelty to the conventional virulence test methods which require tremendous amounts of time and resources. For example, Gnanesh et al. [13] identified a major seedling crown rust resistance gene designated as ‘*PcKM*’ in the oat cultivar ‘Morton’ in field and greenhouse studies; but disease reactions of differential lines and molecular marker data suggested that *PcKM* may be *Pc45*. Another study by Martens et al. [19] and Simons [20] had suggested *Pc54* could be allelic or closely linked with gene *Pc35*; while Leonard et al. [9] placed *Pc35* and *Pc54* on different linkage groups. An association mapping study of elite germplasm identified QTL conditioning crown rust resistance (*Qpc*.*CORE*.*05*) in the same region as *Pc71* [21]. The novelty of these and other QTLs that are effective against crown rust [21,22] could not be determined because of the lack of information about the map location of most *Pc* genes. The current study complements other studies which have mapped crown rust resistance genes, and will improve the availability to oat researchers of information on chromosomal location of *Pc* genes.

The objectives of the present study were to: 1) map *Pc53* on the oat consensus linkage map of Chaffin et al. [23] using SNPs of the 6K Infinium gene chip; and 2) identify SNPs closely linked with *Pc53*.

## Material and methods

### Plant material

A population of 149 F_5_-derived recombinant inbred lines (RILs) was developed from a cross between the *Pc53* differential line, Clinton/6-112-1-15 (Pc53) and the susceptible cultivar Otana, developed by ARS-USDA, Aberdeen, ID (CI 5345/Zanster (CI 5345/2*Overland)). The map location of *Pc53* was validated using 125 F_5_-derived RILs from a cross between the Pc53 and the *Pc50* differential line (Pc50), (CAV 2643/4*Pendek). Both mapping populations were developed by single seed descent at the Small Grains and Potato Germplasm Research Facility of the USDA-ARS in Aberdeen, Idaho.

### Evaluation for crown rust reaction

Seedling tests were carried out on both RIL populations (Pc53 x Otana and Pc53 x Pc50) at the Small Grains and Potato Germplasm Research Facility of the USDA in Aberdeen, Idaho in 2017–2018. *Pca* race LGCG designated according to Chong et al. [24] was used to evaluate populations Pc53 x Otana. The race was virulent against Otana producing infection type (IT) of 3 and 4, but avirulent against Pc53 (infection type; 1). As race LGCG produced similar IT on PC53 and Pc50, a different race ‘NBTG’ was used to evaluate the Pc53 x Pc50 population as it produced distinct IT characterized as small flecks with small uredia (;1) on Pc50, but an immune response (IT of 0) on Pc53. Races ‘LGCG’ and ‘NBTG’ were obtained from the UDA-ARS Small Grains and Potato germplasm Research Facility, Aberdeen, ID and the USDA-ARS Cereal Disease Laboratory, St. Paul, MN, respectively. A single pustule isolate from each of the races was propagated on Otana plants and used to derive inoculum for resistance testing. Approximately, five seedlings from each F_5:6_ RIL, and the parents, were sown in 3.8 cm diameter ‘containers’ containing a 3:2:2 (v:v:v) mix of sand, peat moss, and vermiculite and maintained in a greenhouse adjusted to 22°C and 18 hour photoperiod. Each RIL was planted in three containers, for a total of 15 seed evaluated per line. Two weeks after planting, seedlings were inoculated with uredinia of the respective race suspended in Soltrol170 isoparaffin (Chevron Phillips, The Woodlands, TX) using a motorized sprayer (GAST Manufacturing inc., Benton Harbor, MI) and a small atomizer (G-R Manufacturing Manhattan, KS). Plants were left in the open for one hour to dry, and then transferred to a dew chamber set at 20°C with no light. After 18 hours in the dew chamber, plants were placed in a growth chamber adjusted to 20–22°C with an 18-hour photoperiod. Disease reactions on the first and second leaves were recorded 14 days after inoculation as IT on a 0 to 4 scale where 0–2 are considered resistant reactions, while 3–4 are susceptible reactions [24].

### Genotyping

High quality DNA was isolated from F_5_ plants of the two populations (Pc53 x Otana and Pc53 x Pc50) and parents following a protocol by Anderson et al. [25] with modifications, including grinding freeze dried leaves with beads in a Mixer Mill MM 300 shaker (Retsch, Hannover, Germany) for 10 min at 25 strokes per second. The concentration and quality of DNA were estimated using the BioTek plate reader (BioTek Instruments Inc., Winooski, VT). Genotyping was performed with an Illumina Infinium iSelect oat SNP chip containing 4975 SNPs at the Cereal Crops Research Unit of ARS-USDA in Fargo, ND. Genotype calling for each RIL and parental line was performed automatically using the DBSCAN procedure in GenomeStudio, v. 2.0 (Illumina, San Diego, CA, 2016), and was manually inspected for call accuracy.

### Statistical analysis

Individual RILs in each population were classified based on the IT produced by the F_5:6_ families. The goodness-of-fit of the observed disease reaction to the expected segregation ratio of 1:1 for a single gene was tested using Pearson’s Chi-squared (X^2^) distribution analyses.

### Genetic mapping

SNP markers that were polymorphic between parents of each population, and with less than 10% missing data were selected. Of these, only SNPs that were assigned a location on the oat consensus map of Chaffin et al. [23] were used for analyses. A total of 706 and 569 mapped polymorphic markers were used in the Pc53 x Otana and Pc53 x Pc50 populations, respectively (Table 1). JMP genomics, v. 8.1 (SAS Institute, Cary, NC, 2016) was used to construct genetic linkage maps in both populations. The initial number of linkage groups and marker order in each linkage group were determined using the recombination and linkage groups, and the linkage map order functions of the software, respectively. SNPs were placed into linkage groups using a maximum distance between markers of 30 cM. Genetic distances between markers were calculated in centiMorgans (cM) using the Kosambi map function [26]. Graphical linkage groups were generated using the linkage map viewer function. Linkage groups were assigned to the oat consensus map of Chaffin et al. [23].

**Table 1:** Number of polymorphic SNPs between parental lines and mapped SNPs in the two oat mapping populations.

Population	# SNP (polymorphic)	# SNP (mapped)	Coverage
*Pc53* x Otana	733	706	2276
*Pc53* x *Pc50*	583	569	2223

### Sequence homology

The sequences of the single nucleotide polymorphism markers linked with the *Pc53* gene were compared with the rice (*Oryza sativa* L.) genome (https://www.ncbi.nlm.nih.gov) using the search function ‘sequence(BLAST) search’ to identify candidate genes for *Pc53*.

## Results

### Phenotypic analysis

Race LGCG produced compatible ITs of 3 to 4 on the susceptible parent (Otana) and incompatible ITs of; and; 1 were observed on the resistant parent Pc53. Race NBTG produced ITs of 0 and; 1 on the Pc53 and Pc50 differential lines, respectively. Phenotyping of F_5:6_ families showed that the Pc53 x Otana and Pc53 x Pc50 progeny segregated at a ratio of 79R:71S and 66R:59S. Based on Chi-square tests both populations fit 1R:1S Mendelian ratio with X^2^ of 0.32 and 0.39, and P-value of 0.572 and 0.531, respectively. The segregation ratio shows that the single-seed descent procedure worked as expected without introducing any selection bias.

### Genetic mapping of *Pc53*

**Fig 1:**
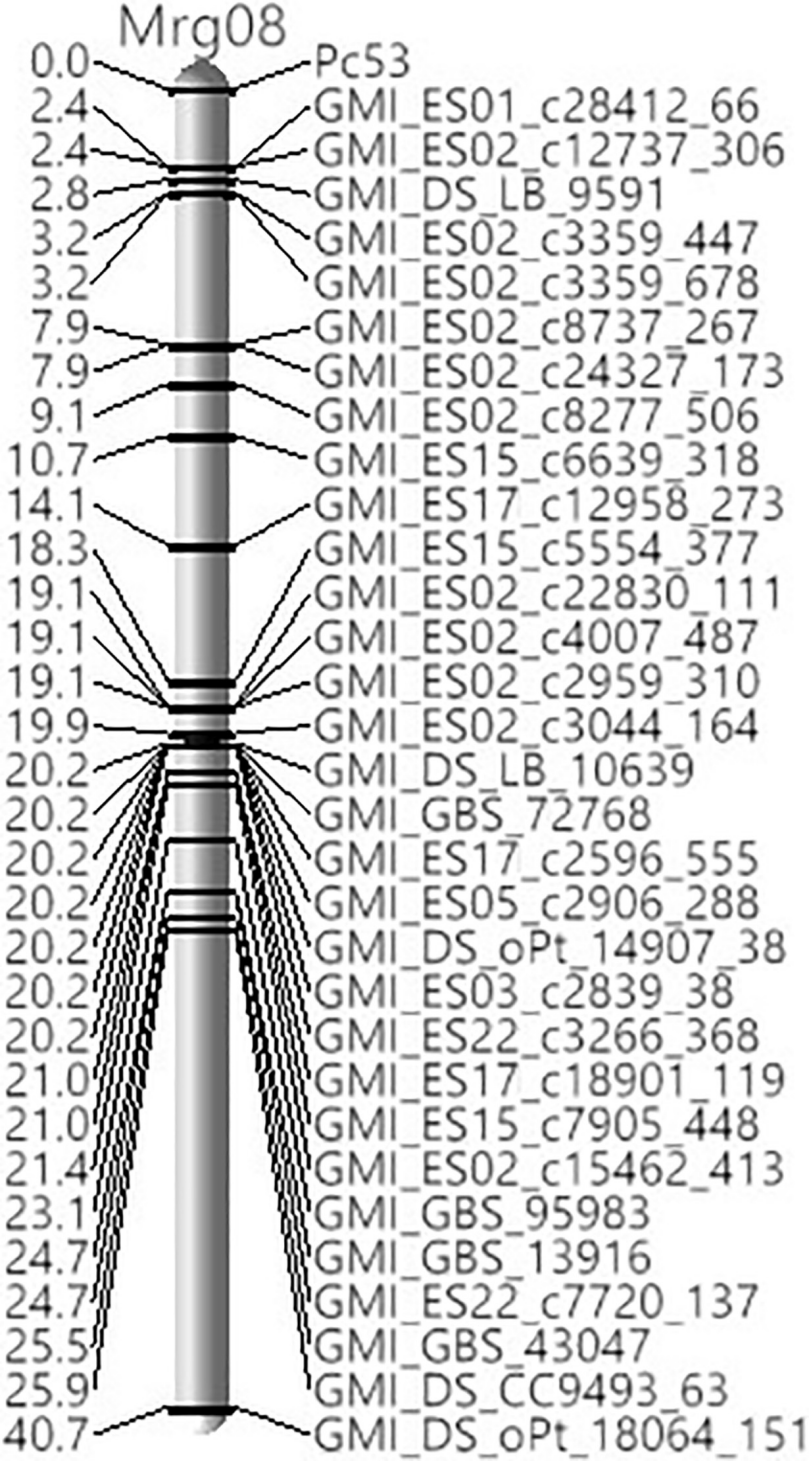
Genetic linkage map of *Pc53* constructed with SNPs and oat F_5:6_ RILs derived from a cross between Pc53 x Otana. The linkage group Mrg08 is based on the oat consensus map of Chaffin et al. [23].

The 706 polymorphic SNP markers were assigned to 18 linkage groups. Linkage analysis revealed that the *Pc53* gene was localized at 2.4 cM distal to the SNP markers GMI_ES01_c28412_66 and GMI_ES02_c12737_306 (Fig 1). Other single nucleotide polymorphism markers closely linked to *Pc53* were GMI_DS_LB_9591 (2.8 cM), GMI_ES02_c3359_447 and GMI_ES02_c3359_678 (3.2 cM), GMI_ES02_c8737_267 and GMI_ES02_c24327_173 (7.9 cM), and GMI_ES02_c8277_506 (9.1 cM). All the SNP markers linked to Pc53 were located on the linkage group Mrg08 of the oat consensus map (23) (Table 2).

**Table 2:** Single nucleotide polymorphism markers closely linked with the crown rust resistance gene *Pc53* in two oat mapping populations. Mrg groups and positions are based on the consensus map developed by Chaffin *et al*. [23].

Population	Marker	Mrg	Position (cM)	Distance from *Pc53* (cM)
Pc53 x Otana	GMI_ES01_c28412_66	8	82.4	2.4 proximal
	GMI_ES02_c12737_306	8	86.2	2.4 proximal
	GMI_DS_LB_9591	8	86.2	2.8 proximal
	GMI_ES02_c3359_678	8	92	3.2 proximal
	GMI_ES02_c3359_447	8	92	3.2 proximal
	GMI_ES02_c8737_267	8	107.1	7.9 proximal
	GMI_ES02_c24327_173	8	109.4	7.9 proximal
	GMI_ES02_c8277_506	8	111.3	9.1 proximal
Pc53 x Pc50	GMI_ES17_c19330_272	8	70.2	9 proximal
	GMI_ES15_c12065_114	8	70.4	9 proximal
	GMI_ES02_c14533_567	8	80.2	0
	GMI_ES15_c3200_563	8	109.2	13.8 distal
	GMI_GBS_821	8	112.5	13.8 distal
	GMI_ES_LB_11757	8	112.5	13.8 distal

### Validation

**Fig 2:**
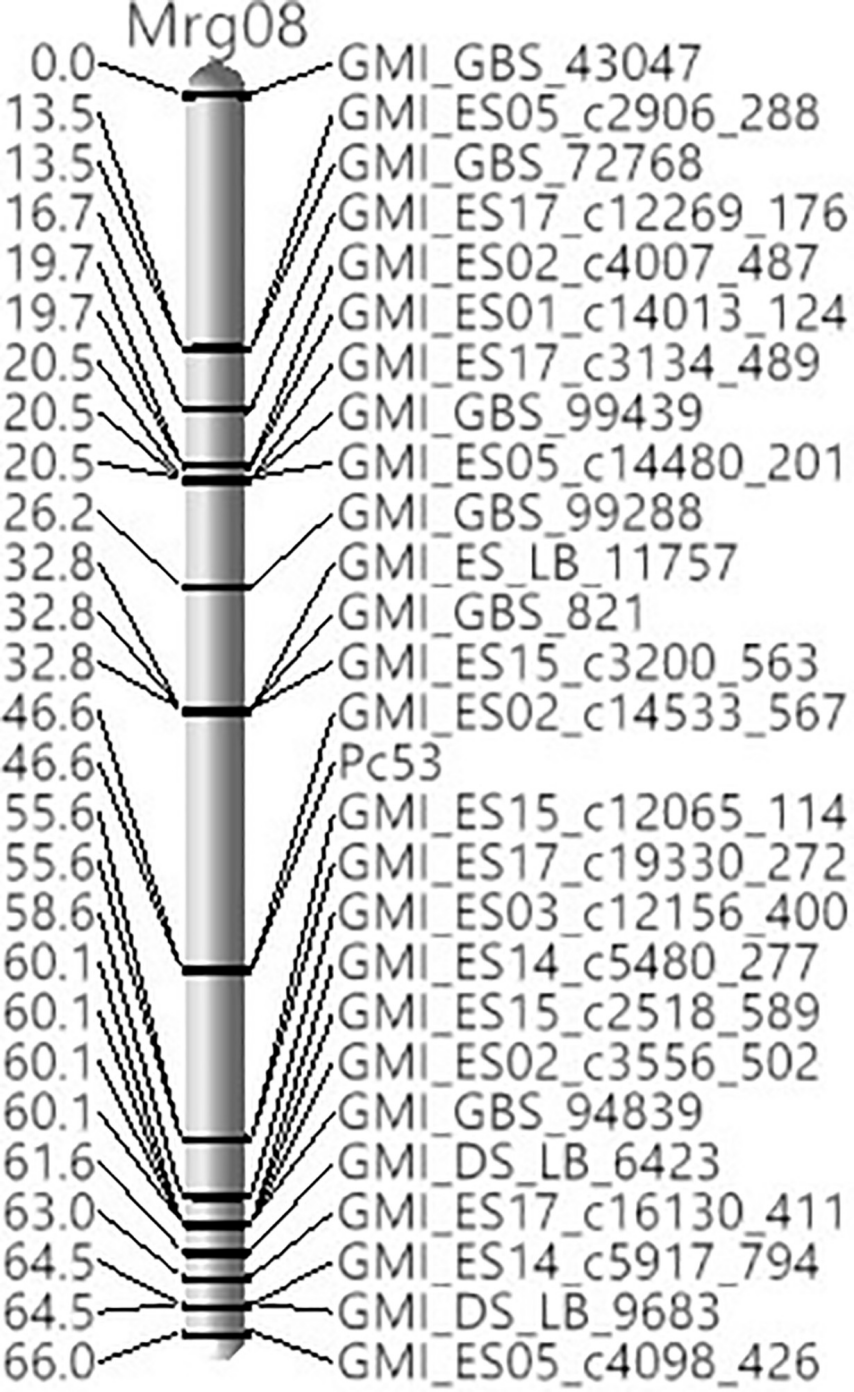
Genetic linkage map of *Pc53* constructed with SNPs and oat F_5:6_ RILs derived from a cross between Pc53 x Pc50. Linkage group Mrg08 is based on the oat consensus map of Chaffin et al. [23].

RILs from the Pc53 x Pc50 population were used to validate the chromosomal location of *Pc53*. The 569 polymorphic markers were assigned to 8 linkage groups. Linkage analysis revealed that *Pc53* was on the same map location as GMI_ES02_c14533_567 at 46.6 cM of the linkage group (Fig 2). SNP markers GMI_ES15_c12065_114 and GMI_ES17_c19330_272 flanked *Pc53* at 9 cM proximal; while GMI_ES15_c3200_563, GMI_GBS_821 and GMI_ES_LB_11757 were 13.8 cM distal to *Pc53*. Like the mapping population the SNP markers linked to *Pc53* were located on linkage group Mrg08 of the oat consensus map [23] (Table 2).

### Sequence homology

According to Oliver et al. [15] Mrg08 of the oat chromosome is homologous to rice chromosome 4. Comparison of the sequences of SNPs closely linked to *Pc53* on Mrg08 of the oat chromosome with the rice genome identified three oat SNPs (GMI_ES02_c14533_567, GMI_ES02_c3359_678, and GMI_ES02_c3359_447) with ≥80% sequence homology to rice loci. Rice chromosome 4 loci LOC107276041 (31764063), LOC_Os04g0661600 (33740410), and LOC_Os04g56620 (33761378) had sequence identity of 90, 80, and 84%, respectively to these three oat SNPs. The former rice locus encodes an uncharacterized protein, and the latter two loci encode the molybdopterin biosynthesis protein CNX1. The oat SNPs closest to the *Pc53* gene in the Pc53 x Otana population (GMI_ES01_c28412_66, GMI_ES02_c12737_306 and GMI_DS_LB_9591) did not have significant sequence homology to a rice locus.

## Discussion

*Pc53* was originally identified from the *A*. *sterilis* accession 6-112-1-15 collected in northern Israel. This gene has not been used in cultivar development apart from some component lines of the Iowa multilines [1]. Virulence to *Pc53* is present in the *Pca* populations of North America [1,10,11]. However, the proportion of isolates collected in a decade (2001–2009) in North America with virulence to *Pc53* has never been greater than 5% of all isolates collected. In contrast, 65–95% of isolates collected in the same period were virulent against the widely deployed genes *Pc38*, *Pc39*, *Pc63*, *Pc67*, and *Pc71* [1]. This result suggests that the *Pc53* gene may have sufficient effectiveness to be useful in variety development, particularly when deployed in combination with other *Pc* genes.

QTL conditioning crown rust resistance in oats have been previously mapped to the Mrg08 linkage group. A major seedling resistance gene ‘*PcKM*’, and the QTL *QCr*.*cdl9-12D* were reported on Mrg08, as were the GWAS QTL *QcC*.*Core*.*08*.*1*, *QCr*.*Core*.*08*.*2*, and *QCr*.*Core*.*08*.*3* [13,21,22]. *QPc*.*Core*.*08*.*2* and *QPc*.*Core*.*08*.*3* were proximal to GMI_ES02_c3359_447 and GMI_ES15_c6639_318, while *Pc53* was distal to both markers, suggesting that the two QTL may not be related to *Pc53*. Similarly, *QCr*.*cdl9-12D* was proximal to GMI_DS_LB_9591, GMI_ES02_c3359_678 and GMI_ES17_c19330_272, and distal to GMI_ES17_c3134_489, GMI_ES01_c14013_124 and GMI_ES17_c12269_176, while *Pc53* was distal to the former three and proximal to the latter three SNPs.

*Pc53* was mapped to within 1 cM of the reported locations of *QPc*.*Core*.*08*.*1* and *PcKM*, suggesting that they may be linked or allelic. Based on crown rust virulence profiles over the period of their field trials, Esvelt-Klos *et al*. [21] suggested *Pc45* and *Pc53* as possible candidate genes for the QTL *QPc*.*Core*.*08*.*1*. The *Pc45* gene has been widely used in North American oat breeding and is expected, therefore, to be abundantly represented in the CORE panel. It is less likely that the QTL identified in the CORE germplasm originated from *Pc53* as this gene is not known to be in the pedigrees of released cultivars apart from the Iowa multi-lines ([1], https://triticeaetoolbox.org/), which were not included in the CORE association mapping panel. In addition, virulence analyses of differential lines and molecular marker data have suggested that *PcKM* might be *Pc45* [13]. To understand the relationship between *Pc45*/*PcKM* and *Pc53* we examined previous virulence analyses reports. Admassu-Yimer et al. [27] reported that *Pca* race ‘MGBH’ produced an IT of 0; on *Pc45* and an IT of 3–4 on *Pc53*. However, the present study placed *Pc53* at the same linkage group and map location as *PcKM* [13]. We hypothesize that *Pc45* and *Pc53* may be components of a complex of genes in this region conditioning resistance to crown rust. Such phenomena are not uncommon in oats. A good example is the resistance of TAM-O-301 to crown rust, which is conferred by complex of *Pc58* genes [12]. Gregory and Wise [28] and Wise *et al*. [29] also reported two and five linked genes respectively conditioning crown rust resistance in diploid oat. In another study by Chong and Brown [30] two to five clustered genes were reported as responsible for crown rust resistance in *A*. *sativa*.

There is currently no sequence available for the *Avena sativa* genome, limiting our ability to exhaustively catalog positional candidate genes for *Pc53*. However, sequence homology of SNPs linked to *Pc53* are consistent with synteny to a region of rice chromosome 4 spanning over 10 Mbp (https://www.ncbi.nlm.nih.gov). A smaller region of synteny corresponding to the region between GMI_ES02_c14533_567 and GMI_ES02_c3359_678 spans close to 2 Mbp and contains over 600 gene annotations. Genes of interest in that region include LOC9269204 disease resistance protein RGA2, and the innate immune system associated LOC4336849 secretory carrier-associated membrane protein 6. In the future, access to an annotated oat genome sequence may enable the identification of positional candidate genes for *Pc53* with sufficient confidence to advance as targets for cloning to further study the mechanisms of crown rust resistance in oats.

In summary, we found that *Pc53* conferring race-specific crown rust resistance in oat is linked with SNP markers placed on the oat consensus linkage group Mrg08. This is the first report of the chromosomal location of *Pc53*. The SNPs linked with it may facilitate the use of this gene in oat breeding programs. In the long term, mapping of oat crown rust resistance genes will contribute towards a more complete understanding of oat genomics and enable researchers to utilize molecular tools to develop new oat cultivars with desirable traits.
